# Match and Training External Load Ratios in Semi-Professional Male Soccer Players: An Exploratory Study

**DOI:** 10.3390/sports14070310

**Published:** 2026-07-22

**Authors:** Sofia Agra, João Barreira, Ricardo Pimenta, Farzad Yousefian, José Afonso, Fábio Yuzo Nakamura

**Affiliations:** 1 Gil Vicente FC Women’s Football Department, 4750-783 Barcelos, Portugal; sofiaagramachado@gmail.com; 2 Research Center in Sports Sciences, Health Sciences, and Human Development (CIDESD), University of Maia, 4475-690 Maia, Portugal; joao_barreira98@outlook.pt (J.B.); rjl.pimenta@gmail.com (R.P.); 3 Research Center of the Polytechnic Institute of Maia (N2i), Maia Polytechnic Institute (IPMAIA), Castêlo da Maia, 4475-690 Maia, Portugal; 4 Department of Rehabilitation and Optimization of Performance (DROP), Futebol Clube de Famalicão-Futebol SAD, 4760-482 Famalicão, Portugal; 5 FSI Lab, Football Science Institute, 18006 Granada, Spain; fyousefi3@gmail.com; 6 Research Center in Sports Sciences, Health Sciences, and Human Development (CIDESD), Department of Sport Sciences, Universidade da Beira Interior, 6201-001 Covilhã, Portugal; 7 Centre of Research, Education, Innovation, and Intervention in Sport (CIFI2D), Faculty of Sport, University of Porto (FADEUP), 4200-450 Porto, Portugal; jneves@fade.up.pt; 8 Graduate Program in Physical Education, Federal University of Pernambuco, Recife 50670-901, Brazil

**Keywords:** football, monitoring, training periodization

## Abstract

This study aimed to quantify and compare the absolute daily and weekly external loads and training-to-match ratios of high-speed running distance (HSRD) and sprint distance (SPD) across the microcycle in semi-professional male soccer players. Twenty-four players from a Portuguese fourth-division team were monitored across nine standardized microcycles (7 days; 1 match) during the 2024–2025 competitive season using Global Navigation Satellite System devices. Absolute HSRD and SPD values were calculated for each training day and match, and daily and weekly training-to-match ratios were derived. Linear mixed-effects models assessed the effects of weekday, playing position, and their interaction. Match day elicited the highest absolute HSRD and SPD across all positions (*p* < 0.001). Within the training week, HSRD peaked on MD-3, whereas SPD peaked on MD-2, with the lowest values observed on MD-1. Significant position-by-day interactions were identified for both HSRD and SPD (*p* < 0.001). Wide defenders and midfielders accumulated greater absolute HSRD during both training and matches, while central defenders exhibited comparatively higher training-to-match ratios despite lower absolute match demands. Notably, in the analyzed team, all playing positions exceeded 100% of match-derived HSRD and SPD during the training week. In conclusion, the semi-professional soccer players investigated herein accumulated weekly high-speed and sprinting loads that exceeded match demands, although their distribution varied by playing position and training day. Confirmatory studies are necessary to further explore training-to-match load ratios across different competitive levels.

## 1. Introduction

Nowadays, soccer training relies on the continuous monitoring of competitive performance and training load, using devices such as Global Navigation Satellite System (GNSS) to help inform load management strategies [1,2,3]. Within contemporary soccer, the increased exposure of players to matches every week [4] resulted in coaches commonly using structured microcycles with consistent weekly training formats (the match-day (MD) approach), while varying the focus of each stimulus to promote player development and recovery [5,6]. For example, in the days following a match (MD + 1 and/or MD + 2), training usually focuses on recovery for players who played more than 60 min, while offering exercises that replicate match demands, such as high-speed running, for those with less or no playing time [7,8]. However, this goal is not always fully achieved [9,10]. On the middle days of the microcycle (MD-3 and MD-4), coaches often plan change-of-direction drills, rondos, small-sided and large-sided games, and collective sectorial tasks, increasing total distance and high-speed running distance (HSRD) covered [11,12]. On the last two days of the microcycle, overall load and volume tend to decrease, with the goal of tapering before the match, and studies often report peak sprint distance and maximal speed exposure being reached on MD-2 [8,11]. Additionally, the number of days between consecutive matches in congested fixture schedules can change how each training day is planned [13,14].

The management of training objectives creates a distribution of training loads that facilitate developing physical fitness while minimizing fatigue as the MD approaches (i.e., the focus is mostly on short-term performance). However, factors such as training philosophy, competitive level, cultural background, and period within the competitive season, among others, might influence the distribution of training loads across the microcycle [5,15]. For instance, Oliveira et al. (2019) [16] found that in a UEFA Champions League team, players exhibited the highest HSRD on MD-5 and MD-4. In contrast, Stevens et al. (2017) [12] reported peak HSRD values on MD-3 for a Dutch Eredivisie team. A survey analysis indicated that MD-3 is the preferred day for high-speed running (HSR) and sprinting (SPR) exercises, particularly in weeks with five or more training sessions. In a study involving Portuguese first, second, and third division teams, Coutinho et al. (2024) [17] demonstrated that load distribution varies by competitive level. For HSRD, teams peaked on MD-3, while the first division team showed a second peak on MD + 1 due to compensatory training for substitute players. For sprint distance (SPD), the first division team peaked on MD-3 and during compensatory training on MD + 1 and MD + 2, the second division team peaked on MD-2, and the third division team peaked on MD + 1. Due to the heterogeneity found across studies and the scarcity of reports on the contribution of each training day relative to match-imposed loads, it is relevant to present data using this approach, especially in competitive levels that are underrepresented in the literature.

Given that MD represents the reference point for soccer training and load management [6,12], several authors have argued that training loads should be expressed relative to match values [11,18]. Accordingly, individual players’ match activity profiles are used to inform daily, weekly, and monthly external loads according to their specific physical demands [18]. This can be done by comparing training loads to the most recent match or a reference set of previous matches (e.g., the five most demanding matches of the season). Nonetheless, findings in the literature are scarce when using such an approach, and the existing evidence is inconsistent. For instance, some studies [19,20] found that no playing position reached 100% of their respective match values for HSR and SPR during microcycle training sessions, with weekly team HSR (>19.8 km/h) loads remaining below match values and resulting in a training-to-match ratio of 0.92. Conversely, Clemente et al. (2019) [21], who monitored a first-division Portuguese team, reported that HSR weekly loads exceeded match values, with the number of training sessions within the microcycle affecting the training-to-match ratios. Similarly, Stevens et al. (2017) [12] reported that HSR training loads exceeded match demands for both professional starters and non-starters.

Considering the variability in the findings reported in the existing literature, this study sought to quantify and compare, at the male semi-professional level, weekly and daily absolute loads and training-to-match ratios of HSR and SPR. Given the limited literature in comparable cohorts, no priori hypotheses are presented for this exploratory study.

## 2. Materials and Methods

### 2.1. Study Design

This observational exploratory study monitored the match and training external workload of 24 male semi-professional soccer players GNSS technology. The sample was determined purposefully but also by convenience criteria (i.e., access to the required data). Data collection was conducted during the 2024–2025 competitive season, between the months of August and April. During this period, only data from 30 training sessions and 9 matches were analyzed, due to the variation in the number of days between matches. Therefore, to ensure consistency, only microcycles in which official matches were played on Sundays were analyzed. Furthermore, only training weeks consisting of five training sessions between matches were included in the study, resulting in a total of nine microcycles (weeks) analyzed. During the observation period, the team played five home and four away matches, with two wins, two draws, and five defeats. It is important to mention that the context of winning, drawing, or losing a match might change the distances covered at high intensity (e.g., draws resulting in higher sprint distance than losses) [22]. Therefore, the fact that the observed sample resulted consisted of more losses might affect match physical performance and the resulting ratios.

### 2.2. Participants

Data were collected from 24 semi-professional male players (age: 24.6 ± 3.5 years; height: 179.2 ± 6.4 cm; weight: 70.6 ± 7.7 kg; senior soccer experience: 6.3 ± 3.5 years) belonging to the same team competing in the Portuguese 4th national division. Players were classified as central defenders (CD, *n* = 4), wide defenders (WD, *n* = 5), midfielders (MF, *n* = 8), wide attackers (WA, *n* = 3), and strikers (S, *n* = 5). It should be noted that the reference team reached the semi-finals of the Portuguese Cup during the 2024–2025 season, competing against clubs from the First Division.

Athletes were excluded from the study if they met any of the following criteria: (1) they were not officially registered for the competition and only participated in training sessions; (2) they did not consistently wear a GPS device during training sessions; or (3) they were undergoing rehabilitation or were injured during the data collection period. In addition, athletes were excluded from the weekly analysis if they: (1) failed to wear the GPS device during any of the training sessions; (2) did not complete at least one training session; (3) sustained an injury at any point during the week; or (4) did not participate in the official match or played less than 60 min. Based on these criteria, six players were excluded from the study. The study involved the analysis of retrospective data collected as part of the team’s routine monitoring procedures. The study was approved by the local ethics committee (#210/2024).

### 2.3. Procedures

All training sessions and matches were monitored using 10 Hz GNSS technology (JOHAN Pacer Trackers, Noordwijk, The Netherlands), which have been previously validated by Nikolaidis et al. [23] to ensure consistent data collection. The GNSS units were activated approximately 20 min prior to the start of each training session and pre-match warm-up to ensure proper satellite connection. While training data included warm-up, training session, and cool-down phases, match data were restricted to effective minutes played (excluding half-time). Data from each device were downloaded and analyzed using the manufacturer’s proprietary software (JOHAN Sync App, version 2.4.0, Noordwijk, The Netherlands). Only valid recordings were retained for analysis, defined as sessions in which GPS satellite signal quality remained stable throughout, and no data loss occurred. Training sessions were conducted on the team’s home pitch during away match weeks and on a synthetic turf field during home match weeks. Official matches were played under standard competition rules on either the team’s home pitch or at official away stadiums.

#### 2.3.1. External Workloads

External loads during official matches and training sessions were quantified using 10 Hz GNSS technology (JOHAN Pacer Trackers, Noordwijk, The Netherlands), which have been previously validated by Nikolaidis et al. [23]. Data from each unit were downloaded and analyzed using the manufacturer’s proprietary software (JOHAN Sync App, version 2.4.0). The external load variables recorded during official matches and training sessions included total distance (TD), high-speed running distance (HSRD; >20.0 km/h), sprint distance (SPD; >25.0 km/h) and number of sprint efforts (SPN; >25.0 km/h), number of acceleration (ACC; >2 m/s^2^) and deceleration (DEC; <−2 m/s^2^) efforts. Only HSRD and SPD were included in analysis of training-to-match ratios. The speed threshold used to define HSRD and SPD are consistent with those adopted by FIFA and UEFA recommendation standards. Although the utilization of arbitrary, non-individualized thresholds is questionable, we adopted them to increase comparability with other studies and focus on our research question.

#### 2.3.2. Training Structure

The physical and tactical components of training were systematically distributed across the five training sessions of the microcycle. The overall structure of the training sessions remained consistent across microcycles, with variation limited to the tactical components in relation to proximity to match day.

Sunday (MD): Match-day.

Monday (MD + 1): The day following MD was a rest day.

Tuesday (MD + 2): The first training session of the week was held two days after the match. During this session, players were divided into two groups (recovery and compensatory) based on their match exposure in the preceding fixture. The recovery group included players who played more than 60 min of the match, and the compensation group included players who played less than 60 min. The aim of the recovery group activities was to facilitate post-match recovery. The compensation group was prescribed activities designed to elicit more than 50% of the team’s mean match values for ACC and DEC efforts. In this study, only data from starting players who participated in recovery sessions were included in the analysis.

Wednesday (MD-4): This session was held four days before the match and was focused on developing strength and power capacities. This session included physical conditioning circuits, ball possession games (20 to 30 m^2^ per player), and transition games (65 to 75 m^2^ per player).

Thursday (MD-3): This session was held three days before the match and was focused on developing endurance capacities and tactical behaviors. Training content included transition games, and collective offensive and defensive organization tasks conducted using 11-vs-11 scenarios on a full-sized pitch.

Friday (MD-2): This session was held two days before the match and was designed to stimulate maximal and near-maximal speed capacities. The session included speed and running technique drills, 40 m sprints, and crossing and finishing exercises. The session ended with a 7-vs-7 (40 m × 33 m) tournament, involving two competing teams while a third team acted as neutral support players positioned on the sidelines.

Saturday (MD-1): This session was held one day before the match and was focused on developing reaction speed and preparing set pieces for the upcoming match.

#### 2.3.3. Data Analysis

To examine the external training load in relation to match demands, both daily and weekly training-to-match ratios were calculated for HSRD and SPD. Daily ratios (HSRD_R_ and SPD_R_) were calculated by dividing the distance covered in each training session (HSRD and SPD) by the corresponding distance covered during the match of the same week. Accordingly, for each training day, the HSRD and SPD distances were expressed as proportions of the respective match value. These ratios provided an index of the extent to which match-derived demands were reproduced within each specific training session. All ratio values were calculated at the individual level and subsequently averaged by playing position and across the entire team.

Weekly training-to-match ratios were calculated by summing the total HSRD and SPD accumulated across all training sessions within a given week and dividing these values by the corresponding match-derived values. Weekly ratios were also calculated for each player and subsequently averaged across playing positions and the entire team. In addition to training-to-match ratios, absolute daily values for HSRD and SPD were also analyzed to characterize the distribution of high-intensity activities throughout the microcycle.

### 2.4. Statistical Analysis

Data normality and homogeneity of variance were assessed using the Kolmogorov–Smirnov and Levene’s test, respectively. Descriptive statistics are reported as mean ± standard deviation (SD). Significance was set at an alpha level of <0.05.

Linear mixed models were implemented to examine differences in external load variables across playing positions and training days. External load metrics were specified as dependent variables, with playing position and weekday (training day) as fixed effects, along with their interaction term (position x training day). Intra-individual variability was accounted for by including player ID as a random effect. Model residuals were visually inspected for normal distribution. When statistically significant main effects were observed, post hoc pairwise comparisons with Bonferroni testing were conducted to assess differences between the training days and playing positions. Test statistics including *t* and F statistics were converted to effect size (ES) using the *effectsize* package for Rstudio (version 2023.12.1) [24]. Cohen’s (*d*) effect sizes and 95% confidence intervals were calculated for each comparison and categorized as trivial (≤0.2), small (>0.2–0.6), moderate (>0.6–1.2), large (>1.2–2.0), very large (>2.0–4.0) and extremely large (>4.0) [25].

## 3. Results

### 3.1. Team and Positional Descriptive Analysis

**Figure 1:**
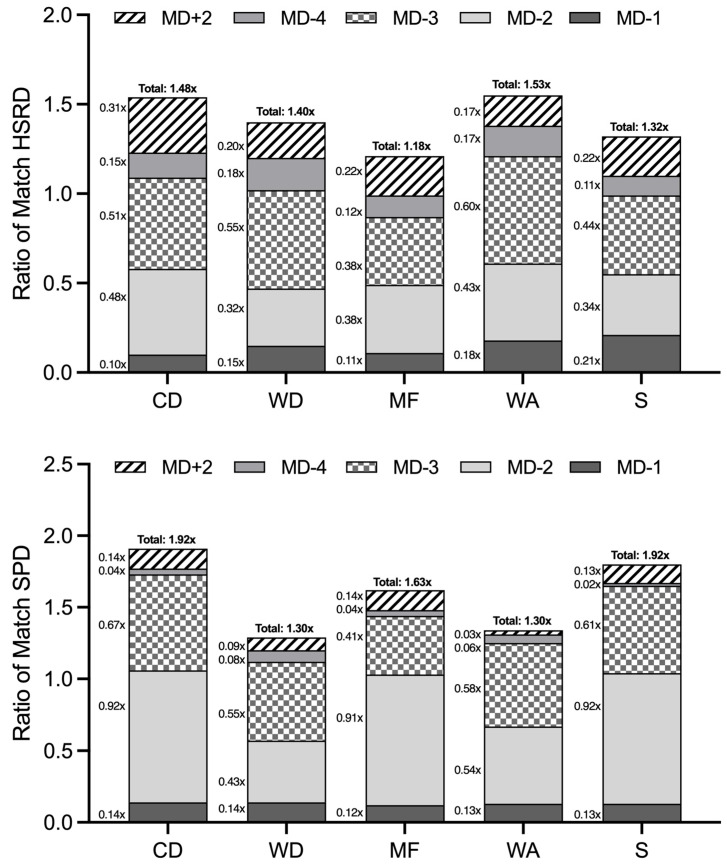
Weekly ratios for HSRD (**upper panel**) and SPD (**lower panel**) by playing position and distribution across training days.

Table 1 presents HSRD and SPD loads accumulated throughout the microcycle and match for the whole team and by playing position. The whole team accumulated greater HSRD during the microcycle, compared to the match. Analysis by playing position showed that WD and WA players also accumulated greater HSRD during the microcycle, compared to the match. Figure 1 presents the total weekly ratio for HSRD and SPRD per position, as well as the ratio in each training day of the microcycle.

### 3.2. Microcycle Distribution of HSR and SPD Ratios

**Figure 2:**
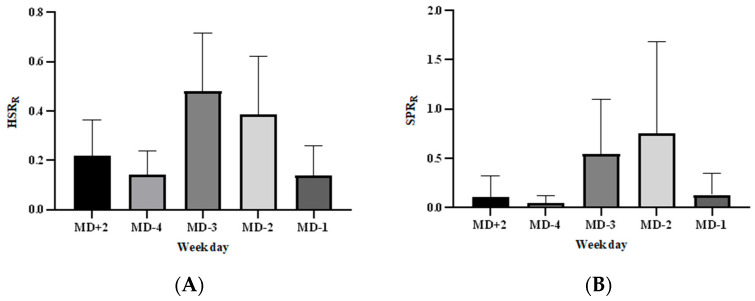
Distribution of HSRD (**A**) and SPD (**B**) ratios for the whole team throughout the training week.

**Figure 3:**
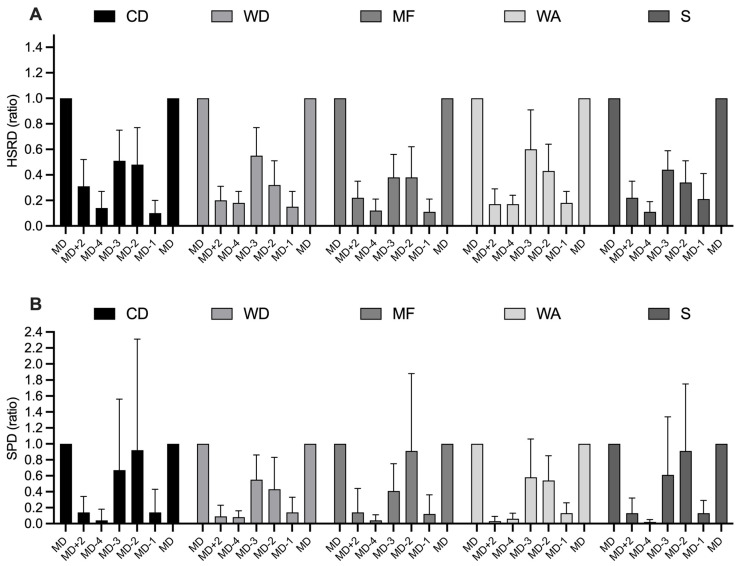
HSRD (**A**) and SPD (**B**) ratio distribution, per position, throughout the microcycle. MD, Match-Day.

Training to match ratios for each day are presented in Figure 2, while weekly ratios by playing position are presented in Figure 3. There was a significant main effect of training day for the training-to-match ratios of both HSRD and SPD. Overall, the highest HSRD ratios occurred on MD-3 and MD-2, whereas MD + 2 and MD-1 displayed the lowest values. A similar pattern was observed for SPD. Ratios on MD-2 were significantly higher than on MD + 2 (+0.65; *p* < 0.001; *d* = −0.39 [−0.49, −0.29]), MD-4 (+0.71; *p* < 0.001; *d* = −0.46 [−0.56, −0.35]), and MD-1 (+0.63; *p* < 0.001; *d* = 0.40 [0.29, 0.50]). SPD on MD-3 exceeded MD + 2 (+0.43; *p* < 0.001; *d* = −0.26 [−0.36, −0.16]), MD-4 (+0.50; *p* < 0.001; *d* = −0.32 [−0.42, −0.22]), and MD-1 (+0.41; *p* < 0.001; *d* = 0.26 [0.16, 0.36]). 

A significant interaction was found between position and training day (*p* < 0.001; Figure 3). Post hoc analyses revealed differences across playing positions. For HSRD ratios, MD-3 was the highest day of the week, exceeding MD-4 across all positions (*p* < 0.05; CD: +0.37; *d* = −0.31 [−0.41, −0.20]; WD: +0.38; *d* = −0.37 [−0.48, −0.26]; MF: +0.26; *d* = −0.34 [−0.44, −0.23]; WA: +0.43; *d* = −0.30 [−0.40, −0.19]; S: +0.32; *d* = −0.21 [−0.31, −0.10]). MD-3 values were higher than MD + 2 for wide attackers (WA; +0.45; *p* < 0.001; *d* = −0.32 [−0.42, −0.21]). HSRD ratios on MD-3 also surpassed MD-1 for CD, WD, MF, and WA (+0.26 to +0.42; *p* < 0.001; *d* = 0.28–0.38). Moreover, MD-2 showed higher HSRD than MD-4 and MD-1 for CD and MF (+0.26 to +0.38; *p* < 0.001; *d* = 0.29 to 0.34). For SPD, significant differences were highlighted in CD and MF, with MD-2 exceeding MD + 2 (+0.77–0.78; *p* < 0.015; *d* = −0.21 to −0.27), MD-4 (+0.87–0.89; *p* < 0.001; *d* = −0.27 to −0.33), and MD-1 (+0.79–0.80; *p* < 0.005; *d* = 0.24 to 0.29).

## 4. Discussion

The aim of this exploratory study was to quantify and compare the absolute weekly and daily external loads and training to match ratios of HSRD and SPD. The results suggest positional differences in the training-to-match ratios as well as positional variation in the peak training-day loads for both variables in a single club’s cohort. All playing positions peaked for HSRD on MD-3. For the SPD variable, only WD peaked on MD-3, while all other positions peaked on MD-2. In both external load metrics, all positions demonstrated overloaded HSRD and SPD demands during the microcycle relative to the match. However, the large number of statistical tests that were performed results in a very large likelihood of false-positive findings; therefore, future confirmatory studies must re-test these parameters to verify whether they hold true in a larger sample of teams.

Distinct patterns in HSRD and SPD were found throughout the microcycles in the investigated team. The highest values observed for HSRD occurred on MD-3, and for SPD, on MD-2. The lowest values observed for HSRD were on MD-1, consistent with the typical tapering approach before match day, and for SPD, on MD-4 [15,21]. Peak HSRD loads observed on MD-3 align with findings of previous studies. Specifically, the studies by Stevens et al. (2017) [12] and Coutinho et al. (2024) [17] were conducted with second- and third-division teams. Coutinho et al. [17] also reported the weekly peak of SPD on MD-2, specifically for a second-division team. While these values are known to be influenced by training philosophy and objectives for each day and week, differences between studies may also exist depending on the data reported. Specifically, some authors present the recovery days (MD + 1 and MD + 2) together with compensatory training data for those days, which can influence the outcomes for the study. In the current study, while it was typical for the reference team to perform compensatory training with the substitute players on MD + 1 or MD + 2, such data was not included in the formal analysis of the current study. Moreover, differences may still exist depending on the strategy and drills used during this training session, as soccer-specific drills may elicit greater acceleration and deceleration frequencies, while isolated running-based drills or the use of larger spaces will induce greater sprinting actions and HSRD [7,18].

Comparing the current findings with previous studies, it seems that MD-1 is the day with the lowest HSRD and SPD loads [12,17,21], which may suggest the implementation of tapering strategies on the day before competition by the reference team. This reduction in volume is likely a strategy to maximize player performance on MD, allowing for optimal recovery and reducing fatigue, which has been associated with an increased risk of hamstring injuries [14,26,27]. Training loads are strategically managed to ensure peak performance during matches, with recovery protocols being crucial in the final days leading to the competition [5,14,28]. In contrast, the day with the lowest SPD and HSRD load in this study was MD-4, which is explained by the type of exercise performed on that day. Given that the primary training drills implemented on MD-4 included strength-oriented workouts, the exercises were all performed in small spaces, preventing the athletes from reaching HSRD or sprinting speeds [29,30]. It is interesting to note that, although this study and the studies by Clemente et al. (2019) [21] and Stevens et al. (2017) [12] were conducted in different contexts, they reported similar values for HSR distance on MD-3. In the current study, the HSRD on MD-3 was 261 m, while other studies reported distances of 247 m and 281 m. Despite the similar values obtained for MD-3, similar trends were not observed for MD-1. In the current study, the HSR distance on MD-1 was 75 m, while other studies reported values above 100 m [12,21]. Training task variability and different methodological approaches implemented during the session before the match, such as longer set-piece training, can likely explain differences between studies and teams.

In our sample, the training-to-match ratio analysis highlights the differential loading tendencies for the CD position. Despite being the position with the lowest weekly accumulated HSRD, along with lowest HSRD exhibited during matches, the CD is among the positions with the highest training-to-match ratio. In contrast, WD presented the highest accumulated HSRD in training and during matches. Interestingly, WD presented lower weekly ratios than CD. This difference likely reflects the different demands of each position in training and is probably a result of position-specific training [19,31]. That is, since soccer training is often focused on match scenarios [1], if a position is unlikely to sprint much during a match, training design and prescription strategies will impose similar demands. On the other hand, for positions often required to perform repeated high-intensity efforts during a match (i.e., wide defenders and attackers), their position-specific training prescription will require the imposition of similar demands. Similarly to the HSRD findings, weekly SPD ratios revealed the highest values were for WA and CD, while the lowest value was observed for WD. However, by comparing the values obtained by WD and WA, the training stimuli did not align with match-specific demands. Although WD accumulated greater SPD during the week, when analyzed in relation to other positions, it presented the lowest training-to-match ratio.

Comparison between the results of the current study with those in the related literature highlights critical discrepancies. It is often reported that soccer players do not typically accumulate external load values, throughout the microcycle, comparable to match-derived demands [19]. For instance, Baptista et al. [19] reported that around 80% of match HSRD and 40% of match SPD is accumulated throughout the training week. Similarly, Miguel et al. [32] reported that around 85% and 40% of match-derived HSRD and SPD is accumulated throughout the training week, respectively. This is a result of both lower accumulated distances in training and higher distances covered during the matches. Interestingly, in our study, all playing positions accumulated greater HSRD and SPD during training, which resulted in ratios greater than 1.0 in relation to the match. This finding may be related to the lower level of competitiveness of our reference team in comparison with the teams investigated in the aforementioned studies, but it is still difficult to define reference values to date. It is important to mention that not only our work but also the majority of studies in the literature addressing the training-to-match load ratios have been employing fixed thresholds to calculate the HSRD and SPD. However, it has been recently argued that there is a need to use individual intensity benchmarks (e.g., critical speed and maximum sprint speed) [3] to calculate training and match loads relative to players’ physiological and mechanical capacities.

### Limitations

This study has several limitations that must be acknowledged. Despite the exploratory nature of the study and known use of ratios among practitioners, calculating ratios has some limitations: first, if ratios are calculated against the previous match, players that did not participate in the match or did not play the whole match cannot be included in the analysis. For this, practitioners may explore other approaches, such as the best 3 matches from the player’s records, matches against the top-ranking teams, the most demanding match, or others. However, when adopting these methodologies, practitioners should also consider factors such as the period of the season those matches took place (i.e., pre-season, previous season) and type of competition (i.e., league or elimination match) as this can have an impact on the outcome. Also, microcycles can vary in the number of training sessions and days between matches. This can greatly influence the accumulated training loads at the end of the training week. We also acknowledge that using fixed thresholds to define HSRD and SRD is a current practice across clubs that may need to be revised in the future through the adoption of thresholds relative to individual physiological and mechanical capacities. Additionally, the low sample size can greatly increase the likelihood of false negatives due to limited statistical power, whereas the high number of statistical tests can increase the likelihood of false positives. As such, future studies should aim at increasing sample sizes and number of observations.

## 5. Conclusions

In this sample of semi-professional male soccer players, weekly high-speed running and sprinting loads accumulated during training exceeded match demands across all playing positions, although their distribution within the microcycle was position- and training day-specific. High-speed running peaked on MD-3 and sprint distance on MD-2, while MD-1 consistently reflected a tapering strategy. Practitioners should carefully monitor the use of predominantly soccer-specific training tasks, as they may differentially expose players to high-intensity running. This can lead to certain positions presenting lower training-to-match ratios despite exhibiting high absolute match demands. Despite the findings, the exploratory nature of the study warrants future confirmatory studies employing larger sample sizes. 

## Figures and Tables

**Table 1:** Descriptive statistics of the accumulated external load during training and match external loads, by playing position.

Playing Position	Metric	Mean ± SD		Difference (Microcycle-Match)	*p*
		Match	Microcycle Accumulated		
Team	HSRD (m)	503 ± 220	720 ± 253	217	<0.001
	SPD (m)	111 ± 77	154 ± 84	43	0.058
CD	HSRD (m)	358 ± 110	491 ± 84	133	0.051
	SPD (m)	77 ± 46	100 ± 57	23	0.413
WD	HSRD (m)	692 ± 141	941 ± 214	249	<0.001
	SPD (m)	186 ± 69	225 ± 83	39	0.101
MF	HSRD (m)	584 ± 188	667 ± 232	83	0.332
	SPD (m)	114 ± 76	144 ± 81	30	0.282
WA	HSRD (m)	360 ± 230	847 ± 226	487	0.001
	SPD (m)	76 ± 66	156 ± 57	80	0.510
S	HSRD (m)	534 ± 141	673 ± 143	139	0.263
	SPD (m)	101 ± 75	124 ± 57	23	0.636

Abbreviations: HSRD, high-speed running distance; SPD, sprint distance.

## Data Availability

Data will be available from the authors upon reasonable request.

## Abbreviations

The following abbreviations are used in this manuscript:

MD	match day
HSRD	high-speed running distance
SPD	sprint running distance
CD	central defender
WD	wide defender
MF	midfielder
WA	wide attacker
S	striker
ES	effect size
GNSS	global navigation satellite systems
